# PARP inhibitors as a radiosensitizer: a future promising approach in prostate cancer?

**DOI:** 10.3332/ecancer.2021.ed118

**Published:** 2021-12-13

**Authors:** Martin Angel, Martin Zarba, Juan Pablo Sade

**Affiliations:** 1 Medical Oncologist, Genitourinary Tumors Department, Instituto Alexander Fleming, Cramer 1180, Ciudad Autonoma de Buenos Aires, C1426ANZ, Argentina; 2 Medical Oncology Fellow, FUCA, Cramer 1180, Ciudad Autonoma de Buenos Aires, C1426ANZ, Argentina; 3 Medical Oncologist, Chief Genitourinary Tumors Department, Instituto Alexander Fleming, Cramer 1180, Ciudad Autonoma de Buenos Aires, C1426ANZ, Argentina; a https://orcid.org/0000-0002-1463-8887

**Keywords:** personalized medicine, radiosensitivity, target therapy

## Abstract

Poly (ADP-ribose) polymerase (PARP) inhibitors (iPARPs) have shown efficacy in homologous recombination (HR) deficiency patients with advanced castration resistant prostate cancer and have shown a radiosensitizing effect in preclinical and early clinical trials. Preclinical data in prostate cancer cells suggest a similar cytotoxic effect with half the radiation dose under the effect of Olaparib or Rucaparib irrespective of HR status. Due to the biologic synergy of radiotherapy (RT) and iPARPs, the risk of recurrence of high-risk prostate cancer and the morbidity associated with prostate cancer local treatment, this interesting strategy seems promising, and a better understanding of the clinical implications remains to be elucidated.

Poly(ADP-ribose) polymerase (PARP) is a DNA repair enzyme which has gained focus in the last decade due to the development of specific inhibitors (iPARPs) [1, 2]. The main role of PARP is to detect single-strand DNA breaks (SSB) and provide specific signals to trigger the enzymatic machinery involved in the SSB repair.

iPARP antitumor activity is based on the concept of synthetic lethality, in which two separate molecular pathways, that are not lethal when disrupted individually, cause cell death when inhibited simultaneously [3–6]. This molecular rational was translated to the clinic since iPARPs showed meaningful benefit in different tumor types, leading to FDA approval for the treatment of advanced ovarian, breast, and more recently, prostate cancer [7–11].

Interestingly, when combined with ionizing radiation, iPARP inhibition has been found to enhance cellular radiosensitivity [12–15]. Radiation induces physical and biochemical DNA damage and cells activate three main repair mechanisms. Two double-stranded DNA breaks (DSB) repair pathways ((non-homologous end-joining, (NHEJ), and homologous recombination, (HR)), and a single-stranded DNA breaks (SSB) repair pathway (base excision repair, BER) [15, 16].

In contrast to the more easily repaired SSB, DSB are highly mutagenic and cytotoxic if unrepaired. However, SSB can be converted to DSB in the context of DNA replication if unrepaired, interfering with important cellular processes and survival. Importantly, SSB are more common in the context of external beam radiotherapy. Therefore, emerging evidence indicates that iPARP can act as radiosensitizers through the BER pathway, increasing the risk of collapsed replication forks, and thus generating persistent DSBs [17].

Furthermore, studies have showed that in addition to HR and NHEJ, DSBs may also be repaired by an alternative end-joining pathway (Alt-EJ), which requires PARP1 [18–20]. Consequently, iPARP use in cells that have switched to Alt-EJ, has demonstrated to enhance radiosensitivity [21].

BRCA1 and BRCA2 proteins are involved in HR repair. The accumulation of SSB that occurs when base excision repair (BER) is malfunctioning, i.e., when PARP is inhibited, produces an increase in DSB due to a failed replication fork that encounters a SSB [22]. These DSBs depend on BRCA proteins to proceed with a DNA conservative repair method (HR). When the BER and HR pathways are inaccessible to cells, they rely on a DNA non-conservative repair mechanism (NHEJ), which lead to genomic instability and cell death [23, 24].

Several iPARP has been investigated in preclinical studies [25]. An in vitro experiment showed a significant increase in DNA damage with the combination of the iPARP olaparib and rucaparib and X-radiation in a neuroblastoma model. Of note, the addition of iPARP allowed to decrease the radiation dose up to 50% without losing its cytotoxic effect [26]. Rae et al, studied the effects of the same iPARPs in combination with radiation in prostate cancer cells. They found a similar cytotoxic effect with half of radiation dose when the cells were exposed to iPARP on both androgen dependent and androgen resistant lines [27, 28].

Clinical evidence on this novel investigational approach consists mainly in phase 1 studies. The first clinical trial exploring combination iPARP and ionizing radiation combines veliparib and whole brain radiation therapy (WBRT) in patients with brain metastases from non-small cell lung cancer. The combination therapy significantly prolongs survival in patient populations with favorable prognosis, but not in patients with unfavorable prognosis. However this benefit was not translated in the phase II trial [14, 29, 30].

In a phase I study of patients with stage II or III rectal cancer, patients were given neoadjuvant capecitabine and radiotherapy in conjunction with escalating doses of veliparib. Maximum tolerated dose was not reached, and the study found 400 mg twice daily to be the appropriate dose of veliparib [31]. The phase II trial showed numerically more patients achieved a pathologic complete response with veliparib (22% vs 34%).

Recently, a phase 1/2 study evaluated the safety of the combination of Olaparib with the radionucleide Ra-223 in pretreated patients with metastatic castrate resistant prostate cancer. The treatment was well tolerated and showed a 6-month rPFS (radiographic progression free survival) of 57%. A phase 2 study is currently recruiting to frther test the hypothesis [32].

High-risk localized prostate cancer is managed with curative intent with surgery or with the combination of radiation therapy plus androgen deprivation therapy. However, approximately half of the patients experience recurrence, with significant prostate cancer-specific morbidity and mortality [33, 34]. Thus, for this group of patients, new treatment options are needed. Recent evidence suggests the existence of a close crosstalk between DNA damage response machinery and androgen hormone signaling pathways mainly based on exposure to ionizing radiation. The AR pathway is activated and transcriptionally upregulates a large subset of DNA repair genes, which improves DNA repair capacity and therefore promotes radio resistance of prostate cancer cells.

To date, scarce clinical evidence supports the benefit of combining iPARP and radiation therapy. This approach may be attractive for different tumor models, including high risk localized prostate cancer which is possibly one of the most suitable scenarios. In this setting, promising phase II trials are on-going, such as the NADIR study investigating niraparib with standard combination radiation therapy and androgen deprivation therapy in patients with high-risk prostate cancer (NCT04037254).

If this first experience shows promising results, it may be the beginning of a new strategy to treat high-risk localized prostate cancer. Currently, this strategy is based on the escalation of radiotherapy doses, on a longer time of ADT and on the intensification of the androgen blockade. Although all these maneuvers have been shown to be more effective, they entail a significant increase in the toxicity associated with the treatment.

## Conclusion

While molecular alterations are currently being explored to make prostate cancer treatment more personalized, defects in DNA repair proteins are rare in localized prostate cancer. Since the potentiation of PARP inhibitors with RT is not based on these germinal or somatic molecular alterations, but on the mechanism of action of RT and ADT, they would be applicable to the majority of patients. To date, this interesting strategy seems promising, and a better understanding of the clinical implications remains to be elucidated.

## Conflicts of interest

None of the authors have conflicts of interest to declare.

## Funding

The authors have not declared a specific grant for this research from any funding agency in the public, commercial or not-for-profit sectors.
